# Essential features of Chiari II malformation in MR imaging: an interobserver reliability study—part 1

**DOI:** 10.1007/s00381-012-1761-5

**Published:** 2012-05-01

**Authors:** Niels Geerdink, Ton van der Vliet, Jan J. Rotteveel, Ton Feuth, Nel Roeleveld, Reinier A. Mullaart

**Affiliations:** 1 Department of Pediatric Neurology 801, Radboud University Nijmegen Medical Centre, P.O. Box 9101, 6500 HB Nijmegen, The Netherlands; 2 Department of Radiology, University Medical Centre Groningen, Groningen, The Netherlands; 3 Department of Epidemiology, Biostatistics and HTA, Radboud University Nijmegen Medical Centre, Nijmegen, The Netherlands

**Keywords:** Chiari II malformation, Spina bifida, Brain MR imaging, Reliability

## Abstract

**Purpose:**

Brain MR imaging is essential in the assessment of Chiari II malformation in clinical and research settings concerning spina bifida. However, the interpretation of morphological features of the malformation on MR images may not always be straightforward. In an attempt to select those features that unambiguously characterize the Chiari II malformation, we investigated the interobserver reliability of all its well-known MR features.

**Methods:**

Brain MR images of 79 children [26 presumed to have Chiari II malformation, 36 presumed to have no cerebral abnormalities, and 17 children in whom some Chiari II malformation features might be present; mean age 10.6 (SD 3.2; range, 6-16) years] were blindly and independently reviewed by three observers. They rated 33 morphological features of the Chiari II malformation as present, absent, or indefinable in three planes (sagittal, axial, and coronal). The interobserver reliability was assessed using *κ* statistics.

**Results:**

Twenty-three of the features studied turned out to be unreliable, whereas the interobserver agreement was almost perfect (*κ* value > 0.8) for nine features (eight in the sagittal plane and one in the axial plane, but none in the coronal plane).

**Conclusions:**

This study presents essential features of the Chiari II malformation on MR images by ruling out the unreliable features. Using these features may improve the assessment of Chiari II malformation in clinical and research settings.

## Introduction

Chiari II malformation is a complex developmental malformation of the central nervous system. It is characterized by a small posterior fossa and downward displacement of the cerebellum and brainstem through an enlarged foramen magnum (hindbrain herniation) [1]. Chiari II malformation is almost uniquely associated with open spinal dysraphism [2]. McLone and Knepper [3] hypothesized that leakage of cerebrospinal fluid through the spinal anomaly reduces the distension of the embryonic ventricular system. The decreased inductive pressure on the surrounding mesenchyme results in an abnormally small posterior fossa. Approximately one third of the patients with Chiari II malformation develop signs and symptoms of brainstem compression [4]. The mortality in this symptomatic group is 15 to 35 % [5, 6].

Usually, Chiari II malformation is clinically diagnosed with the help of MR imaging. On MR images, the malformation is characterized by a constellation of morphological features (Table 1). Most of these features were originally derived from post-mortem examinations [7–10] and computed tomography studies [11–14]. With the introduction of MR imaging, most features were simply adopted to evaluate MR images [15–19]. However, the interpretation of features as seen on MR images may not always be straightforward. First, the malformation is heterogeneous in itself and in its relation with spinal dysraphism. Second, an abundance of features exist, which may obscure unambiguous assessment of Chiari II malformation. Third, the definitions of some features are equivocal and reviewers may interpret features differently. Although most features are typical for Chiari II malformation, knowledge about the reliability of rating these features on MR images is lacking.

**Table 1:** Definitions of features of Chiari II malformation

Feature	Definition	Reference
Sagittal plane		
Downward herniation cerebellum	Either vermis, tonsil, or part of the cerebellum, below the foramen magnum	Variend and Emery [9]
Downward herniation vermis	Vermis below the foramen magnum	Variend and Emery [9]
Downward herniation tonsil	At least one tonsil below the foramen magnum	Variend and Emery [9]
Upward herniation cerebellum	Bulging of the cerebellum through the tentorial incisura	Peach [7] and Naidich et al. [12]
Downward displacement medulla	Stretching and downward displacement of the medulla below the foramen magnum	Emery and MacKenzie [8]
Downward displacement pons	Stretching and downward displacement of the pons towards spinal canal	Naidich et al. [14]
Downward displacement fourth ventricle	Stretching and downward displacement of the fourth ventricle	Emery and MacKenzie [8]
Medullary kinking	Kink of the medulla dorsal to the upper cervical spinal cord	Emery and MacKenzie [8]
Flattened pons	Thin stretched pons	El Gammal et al. [16]
Abnormal width fourth ventricle	Collapsed or dilated fourth ventricle	Wolpert et al. [15]
Hypoplastic tentorium	Underdeveloped tentorium with abnormally low insertion at the occipital bone	Peach [7] and Naidich et al. [11]
Abnormal course straight sinus	Abnormally short, steep course, or low insertion of the straight sinus	El Gammal et al. [16] and Just et al. [17]
Beaked tectum	Deformity of the quadrigeminal plate appearing like a pointed or bulbous mass	Peach [7] and Wolpert et al. [15]
Enlarged massa intermedia	Thick interthalamic adhesion	Peach [7] and Naidich et al. [13]
Stenogyria	Innumerable, closely spaced small gyri at the occipital cortex	Peach [7] and Wolpert et al. [15]
Axial plane		
Cerebellum in cervical spinal canal	Cerebellum below the top of the dens or the base of the occipital condyles	Variend and Emery [9]
Vermis in cervical spinal canal	Vermis below the top of the dens or the base of the occipital condyles	Variend and Emery [9]
Tonsil in cervical spinal canal	At least one tonsil below the top of the dens or the base of the occipital condyles	Variend and Emery [9]
Cerebellum wrapped around brainstem	Cerebellar hemispheres wrapped around brainstem into cerebellopontine angle cisterns	Peach [7] and Naidich et al. [12]
Abnormal fissural pattern of cerebellum	Abnormal fissural and lobular pattern of the superior surface of the cerebellum	Variend et al. [10]
Small fourth ventricle	Collapsed fourth ventricle	Wolpert et al. [15]
Enlarged fourth ventricle	Dilated fourth ventricle	Wolpert et al. [15]
Beaked tectum	Quadrigeminal plate is stretched appearing beaked	Peach [7] and Naidich et al. [12]
Enlarged massa intermedia	Thick interthalamic adhesion	Peach [7] and Naidich et al. [13]
Gyral interdigitation	Gyri crossing the interhemispheric fissure and folding in contralateral sulci	Peach [7] and Just et al. [17]
Stenogyria	Innumerable, closely spaced small gyri at the occipital cortex	Peach [7] and Wolpert et al. [15]
Coronal plane		
Downward herniation cerebellum	Cerebellum below the base of the occipital condyles	Variend and Emery [9]
Downward herniation vermis	Vermis below the base of the occipital condyles	Variend and Emery [9]
Downward herniation tonsil	At least one tonsil below the base of the occipital condyles	Variend and Emery [9]
Upward herniation cerebellum	Upward bulging of the cerebellum (towering) through a wide tentorial incisura	Peach [7] and Wolpert et al. [15]
Indentation	Indentation of the cerebellum by the edge of the tentorium	Peach [7] and Naidich et al. [12]
Hypoplastic tentorium	Short tentorial leaves with a wide tentorial incisura	Peach [7] and Naidich et al. [11]
Gyral interdigitation	Gyri crossing interhemispheric fissure and folding in contralateral sulci	Peach [7] and Just et al. [17]

Still, brain MR imaging plays a substantial role in clinical decision making regarding the management of children with spina bifida [18, 20]. On the one hand, the discussion on selective treatment of severely affected newborn infants is still ongoing [21]. On the other hand, fetal imaging and prenatal surgery are becoming more important every day. Recently, a randomized control trial showed important improvement of hindbrain herniation following prenatal surgery for spina bifida [22]. However, the assessment of Chiari II malformation may be even more complicated in prenatal MR imaging. A discrepancy of 41 % was seen in judgment of the degree of cerebellar herniation in prenatal MR imaging studies [23]. When choices have to be made about pre- and postnatal treatment options, it is important to have consensus about the morphological features that unambiguously characterize Chiari II malformation. As a proper reference standard is not available, however, testing the validity of different features is unattainable. The next best method to appraise these features is to evaluate interobserver reliability.

Therefore, we initiated a study to investigate the interobserver reliability of morphological features of Chiari II malformation on MR images. The purpose of this study was to select those features among the abundance of features that are essential for the diagnosis of the malformation, hypothesizing that several features would be too unreliable to adequately characterize Chiari II malformation.

## Material and methods

### Patients

Brain MR images of 79 children [mean age 10.6 (SD 3.2; range, 6-16) years] were evaluated. Of these children, 26 had open spinal dysraphism, while 17 children had closed spinal dysraphism (13 with lipomyelomeningocele and four children with other types of closed spinal dysraphism). The children with open spinal dysraphism were presumed to have Chiari II malformation [2], while children with closed spinal dysraphism might have some features of hindbrain herniation according to the literature [24, 25]. The latter group was included to reduce context bias [26]. The majority of these children with spinal dysraphism (*n* = 36) were recruited at the outpatient clinics of Pediatric Neurology of the Radboud University Nijmegen Medical Centre (RUNMC) as part of a prospective research program dedicated to outcome and prognosis of spina bifida. MR images of the remaining seven children were obtained retrospectively from the archives of the Department of Radiology of the RUNMC, from which we also obtained MR images of 36 children without spinal dysraphism, who were presumed to have no cerebral pathology. Although MR imaging in these 36 children was performed with suspicion of or to rule out cerebral pathology, the images had been assessed as normal by an independent radiologist in a clinical setting before the start of the study.

### MR imaging

All MR images were acquired using a 1.5-T MR imaging unit (Siemens Avanto; Siemens Medical Solutions, Erlangen, Germany) with a standard head coil. MR imaging in the 36 children who were part of the prospective research program consisted of T1-weigthed images in the sagittal plane and T2-weigthed images in the axial and coronal plane. The retrospectively obtained MR images were acquired using comparable sequences. For different reasons, MR images were not acquired in three planes for all 79 children. Images in the sagittal plane were available for 69 children (41 with spinal dysraphism), images in the axial plane for 58 children (32 with spinal dysraphism), and images in the coronal plane for 51 children (37 with spinal dysraphism).

The Regional Committee on Research involving Human Subjects approved the study protocol. Prior to inclusion in the study, written informed consent was obtained from the parents of all 36 children and all children above 12 years of age taking part in the prospective research program.

### Image analysis

All MR images were blinded for demographic and diagnostic information. The MR images were mixed and arranged by plane into three data sets: a sagittal set, an axial set, and a coronal set. These three data sets were reviewed consecutively and independently by three observers: a junior pediatric neurologist (N.G.) with 6 years of experience in reviewing pediatric brain MR images, a senior pediatric neurologist (R.A.M.), and a senior neuroradiologist (T.V.), both with more than 20 years of experience in reviewing pediatric brain MR images. A few weeks separated the reviews of the three datasets to prevent bias by recognition of images from a former set as much as possible. The images were available on compact disks and were reviewed on an Agfa workstation or on a personal computer using Agfa software (Impax Client, release 4.5).

The morphological features of Chiari II malformation to be assessed were selected from the literature and incorporated in a review protocol (Table 1). First, the feasibility of the protocol was evaluated in a pilot study (*n* = 10), resulting in a final set of study features with their definitions. The observers rated all features as being present, absent, or indefinable.

### Statistical analysis

For each feature, the ‘present’, ‘absent’, and ‘indefinable’ ratings were tallied up per observer. First, the ‘indefinable’ ratings were evaluated to assess the applicability of each feature. If two or three observers rated a feature as indefinable in more than 5 % of the MR images, it was qualified as non-applicable and subsequently excluded from the further analyses.

Interobserver agreement analyses were performed for the applicable features using only the ‘present’ and ‘absent’ ratings. The percentages of agreement were obtained from contingency tables. Based on these tables, *κ* values for multiple observers were calculated to measure the extent of agreement among the three observers [27]. To comprehend possible sources of disagreement, *κ* values were also calculated for pairs of observers. We considered a feature reliable when the *κ* value was above 0.8, which denotes almost perfect agreement [28]. The analyses were performed using SAS software version 8.2 (SAS Institute).

## Results

For each feature, the percentages of ‘present’ and ‘indefinable’ ratings are summarized per observer in Table 2. All observers rated most features in the sagittal plane as present in 20–35 % of the MR images, whereas the percentages of ‘present’ ratings in the axial and coronal planes varied substantially among features and among observers. In general, observer C rated features as ‘present’ less often than the other two observers did, whereas observer B rated features as ‘indefinable’ more often than the other two observers did. In the sagittal plane, all but one feature (*Stenogyria)* turned out to be applicable. In contrast, in the axial and coronal plane more than half of the features turned out to be non-applicable (Table 2). One observer rated *Enlarged massa intermedia* in the axial plane as indefinable in all but one MR image. The ratings of features in children with open or closed spinal dysraphism or without spinal dysraphism are presented in Table 3. With a few exceptions, features were quite common in children with open spinal dysraphism and hardly seen in the other children.

**Table 2:** Proportions of ‘present’ and ‘indefinable’ ratings per observer for each feature of Chiari II malformation

Feature	Present			Indefinable			Non-applicable^a^
	A	B	C	A	B	C	
Sagittal plane							
Downward herniation cerebellum	35	33	35	−	−	−	
Downward herniation vermis	25	28	35	3	3	−	
Downward herniation tonsil	33	30	26	1	1	6	
Upward herniation cerebellum	13	17	6	−	6	3	
Downward displacement medulla	30	26	20	−	−	3	
Downward displacement pons	26	26	13	−	3	3	
Downward displacement fourth ventricle	25	23	20	−	4	1	
Medullary kinking	17	14	14	1	1	6	
Flattened pons	25	38	23	−	−	−	
Abnormal width fourth ventricle	25^b^	25	29^b^	−	−	1	
Hypoplastic tentorium	26	22	22	−	13	3	
Abnormal course straight sinus	23	23	29	9	4	3	
Beaked tectum	25	28	23	−	−	−	
Enlarged massa intermedia	43	62	10	−	−	4	
Stenogyria	19	7	9	3	22	12	+
Axial plane							
Cerebellum in cervical spinal canal	21	21	19	10	19	12	+
Vermis in cervical spinal canal	2	2	14	26	36	16	+
Tonsil in cervical spinal canal	7	5	16	24	34	16	+
Cerebellum wrapped around brainstem	29	24	3	−	5	2	
Abnormal fissural pattern of cerebellum	29	59	47	7	7	5	+
Small fourth ventricle	26	28	26	−	3	−	
Enlarged fourth ventricle	3	2	3	−	9	−	
Beaked tectum	19	26	19	7	7	7	+
Enlarged massa intermedia	17	12	−	−	3	98	+
Gyral interdigitation	22	31	17	5	7	5	+
Stenogyria	17	9	7	7	91	12	+
Coronal plane							
Downward herniation cerebellum	35	26	24	8	8	4	+
Downward herniation vermis	10	69	14	18	31	6	+
Downward herniation tonsil	35	24	24	8	10	2	+
Upward herniation cerebellum	26	12	8	2	6	6	+
Indentation	12	12	6	2	4	−	
Hypoplastic tentorium	26	2	14	4	61	2	
Gyral interdigitation	18	26	14	2	10	4	

Data are percentages

*A* observer A, *B* observer B, *C* observer C

^a^At least two observers considered the feature as indefinable in more than 5 % of the MR images

^b^All abnormally small fourth ventricles, except for one dilated fourth ventricle

**Table 3:** Features of Chiari II malformation present on MR images in children with open or closed spinal dysraphism or without spinal dysraphism

Feature	Spinal dysraphism		No spinal dysraphism
	Open	Closed	
	(%^a^)	(%^a^)	(%^a^)
Sagittal plane (*n* = 207)			
Downward herniation cerebellum	83	16	4
Downward herniation vermis	74	10	2
Downward herniation tonsil	75	14	1
Upward herniation cerebellum	33	2	0
Downward displacement medulla	68	8	0
Downward displacement pons	61	2	0
Downward displacement fourth ventricle	64	2	0
Medullary kinking	40	6	0
Flattened pons	75	2	5
Abnormal width fourth ventricle	74	2	0
Hypoplastic tentorium	67	0	0
Abnormal course straight sinus	71	0	7
Beaked tectum	72	0	0
Enlarged massa intermedia	36	35	43
Stenogyria	32	2	0
Axial plane (*n* = 174)			
Cerebellum in cervical spinal canal	47	7	3
Vermis in cervical spinal canal	15	0	0
Tonsil in cervical spinal canal	24	0	0
Cerebellum wrapped around brainstem	50	0	0
Abnormal fissural pattern of cerebellum	68	43	26
Small fourth ventricle	68	3	0
Enlarged fourth ventricle	8	0	0
Beaked tectum	56	0	0
Enlarged massa intermedia	23	0	3
Gyral interdigitation	56	7	3
Stenogyria	21	0	0
Coronal plane (*n* = 153)			
Downward herniation cerebellum	67	6	0
Downward herniation vermis	65	6	0
Downward herniation tonsil	20	0	0
Upward herniation cerebellum	38	0	0
Indentation	25	0	0
Hypoplastic tentorium	35	0	0
Gyral interdigitation	38	12	0

^a^The numbers represent percentages of present ratings based on the overall ratings of three observers

**Fig. 1:**
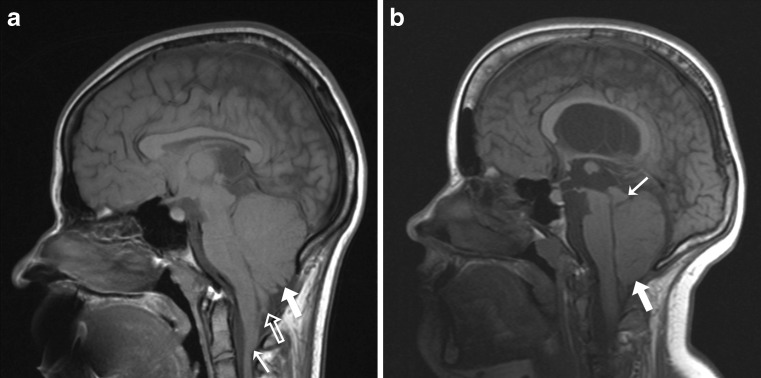
**a** Sagittal T1-weighted brain MR image in 16-year-old child with open spinal dysraphism. The image shows herniation of the vermis (*large white arrow*), herniation of the tonsil (*large white open arrow*), and medullary kinking (*small white arrow*); **b** sagittal T1-weighted brain MR image in 12-year-old child with open spinal dysraphism. The image shows herniation of the cerebellum (*large white arrow*). The vermis and tonsil cannot be demarcated from each other. Note the beaked tectum (*small white arrow*) and the hypoplastic tentorium. Also, note the downward displacement of the medulla and pons and the small fourth ventricle in both images

The interobserver agreement of the applicable features is presented in Table 4. The right panel of the table shows the percentages of agreement and disagreement, while the left panel shows the *κ* values. The interobserver agreement among all three observers was almost perfect (*κ* value > 0.8) for the following features in the sagittal plane: *Downward herniation cerebellum*, *Downward herniation tonsil*, *Downward displacement medulla*, *Downward displacement fourth ventricle*, *Medullary kinking*, *Abnormal width fourth ventricle*, *Hypoplastic tentorium*, and *Beaked tectum* (Fig. 1). Only one feature in the axial plane (*Small fourth ventricle*) showed almost perfect agreement, while none of the features in the coronal plane did. The overall *κ* values for the remaining features ranged from 0.50 (*Cerebellum wrapped around brainstem*) to 0.75 (*Downward displacement pons*), except for a very low *κ* value for *Enlarged massa intermedia* (0.10). Table 4 also lists the *κ* values for pairs of observers. For seven features, the *κ* values differed substantially among pairs of observers: *Downward herniation vermis*, *Upward herniation cerebellum*, *Downward displacement pons*, and *Abnormal course straight sinus* in the sagittal plane; *Cerebellum wrapped around brainstem* in the axial plane; and *Indentation* and *Gyral interdigitation* in the coronal plane. In general, the agreement between observers A and B was stronger than the agreement of each of them with observer C.

**Table 4:** Overall and pairwise interobserver agreement of the applicable features of Chiari II malformation

Feature	*κ* value				Agreement (%)		Disagreement (%)	No.
	Overall	Pairwise			All rated	All rated		
		A–B	A–C	B–C	‘Present’	‘Absent’		
Sagittal plane								
Downward herniation cerebellum	*0.85*	0.90	0.87	0.77	29	61	10	69
Downward herniation vermis	0.72	0.84	0.66	0.67	20	63	17	66
Downward herniation tonsil	*0.85*	0.85	0.89	0.80	24	67	9	64
Upward herniation cerebellum	0.56	0.66	0.57	0.42	5	82	13	63
Downward displacement medulla	*0.83*	0.88	0.83	0.78	19	72	9	65
Downward displacement pons	0.75	0.96	0.64	0.60	12	76	12	65
Downward displacement fourth ventricle	*0.84*	0.87	0.85	0.80	17	75	8	65
Medullary kinking	*0.88*	0.94	0.88	0.81	12	83	5	64
Flattened pons	0.70	0.70	0.72	0.67	19	62	19	69
Abnormal width fourth ventricle	*0.85*	0.92	0.81	0.81	21	70	9	67
Hypoplastic tentorium	*0.84*	0.86	0.76	0.91	19	73	8	60
Abnormal course straight sinus	0.73	0.85	0.61	0.75	17	71	12	55
Beaked tectum	*0.90*	0.92	0.88	0.89	22	72	6	69
Enlarged massa intermedia	0.10	0.27	0.00	0.03	4	26	70	66
Axial plane								
Cerebellum wrapped around brainstem	0.50	0.95	0.18	0.20	4	73	23	55
Small fourth ventricle	*0.85*	0.87	0.91	0.78	23	68	9	56
Enlarged fourth ventricle	–^a^	–^a^	–^a^	–^a^	2	98	0	53
Enlarged massa intermedia	–^a^	–^a^	–^a^	–^a^	0	100	0	1
Coronal plane								
Indentation	0.70	0.81	0.63	0.63	6	86	8	48
Hypoplastic tentorium	–^a^	–^a^	–^a^	–^a^	5	85	10	20
Gyral interdigitation	0.63	0.61	0.76	0.54	11	71	18	44

Overall *κ* value > 0.8 indicating almost perfect agreement are presented in italics *A* observer A, *B* observer B, *C* observer C

^a^κ value could not be calculated, because one or more counts were too small

## Discussion

On brain MR images, Chiari II malformation is generally assessed based on a constellation of morphological features. The current study reports on the reliability of these features leading to the identification of essential features that may improve consensus on the diagnosis of Chiari II malformation.

In this study, reliable features were distinguished from unreliable features, with reliable features predominantly being found in the sagittal plane. This in itself is not surprising, as most of the morphological abnormalities are best shown in the midsagittal plane, which is usually used to assess Chiari II malformation. Still, a substantial number of features in the sagittal plane (six out of 14) showed less than perfect or poor reliability and most features in the axial and coronal plane were non-applicable. These results support our assumption that the MR interpretation of Chiari II malformation is not always straightforward. The unreliability of features may be explained by their qualitative nature and the fact that the distinction between normal and abnormal brain development is not defined by an unambiguous cutoff point. Judgment of the features is further complicated by the morphological diversity of the malformation and the fact that MR images capture features to various degrees. These general explanations mainly apply to features with random disagreement, that is to say, when the overall *κ* value and all pairwise *κ* values are low (e.g., *Upward herniation cerebellum*, *Flattened pons*, and *Gyral interdigitation*; Table 4).

**Fig. 2:**
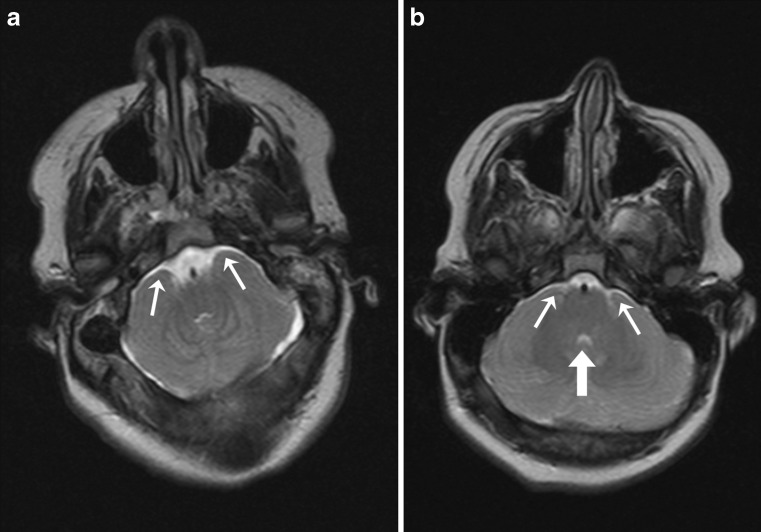
**a** Axial T2-weighted brain MR image in 16-year-old child with open spinal dysraphism. The image clearly shows that the cerebellar hemispheres are wrapped around the brainstem (*small white arrows*); **b** axial T2-weighted brain MR image in 12-year-old child with open spinal dysraphism. In this image, it is questionable whether the cerebellar hemispheres are wrapped around the brainstem (*small white arrows*). Also note the small fourth ventricle (*large white arrow*)

**Fig. 3:**
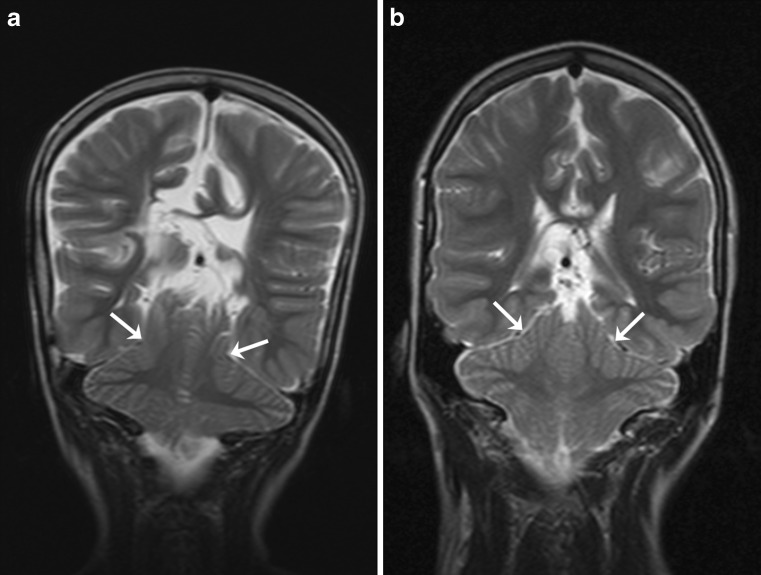
**a** Coronal T2-weighted brain MR image in 9-year-old child with open spinal dysraphism. The image clearly shows that the tentorium indents the cerebellar hemispheres (*white arrows*); **b** coronal T2-weighted brain MR image in 12-year-old child with open spinal dysraphism. In this image, it is questionable whether the tentorium indents the cerebellar hemispheres (*white arrows*)

On the other hand, the results for pairwise agreement showed systematic disagreement for some features; i.e., stronger agreement between observers A and B than the agreement for each of them with observer C. Perhaps, reappraisal of some definitions may further improve reliability, for instance, for *Cerebellum wrapped around brainstem* and *Indentation* (Figs. 2 and 3).

The systematic disagreement for *Downward herniation vermis* is of special interest. Blurred cerebellar contours in a crowded posterior fossa and partial volume effects may hamper precise demarcation of the vermis and may make it difficult to distinguish the vermis from the tonsil and from medullary kinking (Fig. 1). This is in agreement with previous studies that reported that the vermis could not be clearly delineated in about 50 % of children with Chiari II malformation [15, 16]. On the other hand, systematic disagreement may have resulted from different concepts about the morphology of Chiari II malformation. Observer C, in contrast to the other two observers, considered *Downward herniation vermis* to be present more often than *Downward herniation tonsil* (Table 2). Yet, from post-mortem studies, it is known that herniation of the vermis without herniation of the tonsils does not occur [9]. Therefore, we recommend to assess downward herniation of the cerebellum irrespective of this being herniation of the vermis or herniation of the tonsils.

One of the limitations of this study was the possibility of context bias, i.e., knowledge from other sources that exaggerated interobserver agreement [26]. To deal with this phenomenon, we mixed the images expected to show Chiari II malformation with images expected to be without abnormalities and with images in which some features of hindbrain herniation could be present. However, observers may have tended to rate a feature according to the general appearance of the cerebellum, as complete blinding of each solitary feature was impossible. Another potential source of bias was the ratio between present and absent ratings as excess of one of the two affects the *κ* value [29]. In the current study, the proportion of present ratings per feature generally ranged from 25 to 35 % (Table 2). Within this small range, *κ* values can be safely compared among features. Yet, a few features were rated as present in considerably lower proportions. As the *κ* value will underestimate agreement in case of low proportions [29], reliability of the features in question may be better than expected from the actual *κ* values. Furthermore, response bias may have decreased *κ* values [29, 30]. This is particularly relevant when a rating is ambiguous. Although the observers had the opportunity to rate ambiguous features as indefinable, response bias was not completely avoided, since observers A and B generally rated features more often as present than observer C. As this was clearly the case for *Downward displacement pons* and the *κ* value was just below the cutoff point of 0.8, underestimation of agreement may be relevant for this feature. Potential institutional bias may be another limitation of the study. All observers worked at the same academic hospital, which might have increased agreement. However, the observers differed in terms of experience and educational and professional background. These differences might have reduced the interobserver agreement. On the other hand, the participation of senior and junior specialists with different backgrounds implies that the results are particularly useful for radiologist and other specialists who might be less familiar with reviewing brain MR images.

Nevertheless, this study showed that among all features that are evaluated while diagnosing Chiari II malformation, only a subset seems to be reliable. Although the Chiari II malformation seems to be a clear entity, clinicians and researchers should be aware of the different interpretations of its features among observers. The use of reliable features may facilitate plain communication about Chiari II malformation in clinical and research settings. In the management of individual patients, decisions about treatment options should be based on clinical signs and symptoms in combination with reliable MR findings. Although Chiari II malformation is almost uniquely associated with open spinal dysraphism, there might be exceptions. In such cases, the reliable features presented might be useful. In discussions on prenatal surgery and postnatal selective treatment of spina bifida, this study provides clinicians and researchers with features that unambiguously describe the Chiari II malformation.

In addition to the qualitative method, a morphometric approach quantifying the morphological distortions may be helpful to overcome the problems of unreliable features. Morphometric measures are less subjective and may be less liable to interobserver variability. They may also provide cutoff points that distinguish between normal and abnormal brain development. The reliability and diagnostic performance of morphometric measures is subject of the second part of our study on MR assessment of Chiari II malformation.

In conclusion, the following morphological features can reliably be used to assess Chiari II malformation on MR images: downward herniation of the cerebellum, downward displacement of the medulla, pons, and fourth ventricle, medullary kinking, abnormally shaped fourth ventricle, hypoplastic tentorium, and beaked mesencephalic tectum. The use of these essential features may improve MR assessment of Chiari II malformation by providing a solid basis for consensus on the diagnosis.
